# Development and Validation of the Approach-Avoidance System Questionnaire (AASQ)

**DOI:** 10.3389/fpsyg.2019.02531

**Published:** 2019-11-14

**Authors:** Anne Teboul, Cyril Klosek, Camille Montiny, Christophe Gernigon

**Affiliations:** Laboratory Epsylon, Faculty of Sport and Physical Education Sciences, University of Montpellier, Montpellier, France

**Keywords:** achievement motivation, achievement goals, attractors, dynamical systems, competence, self

## Abstract

Gernigon et al. (2015) recently proposed a dynamical model of approach and avoidance, according to which approach and avoidance patterns are accounted for by a control parameter *k*, which results itself from the interactions among a limited number of key social-cognitive variables: competence expectancies (*c*), benefit for the self (*b*_*s*_), and threat for the self (*t*_*s*_). The present research aimed to develop and validate a French questionnaire that measures these variables, the Approach-Avoidance System Questionnaire (AASQ). A first study revealed a satisfactory 3-factor structure based on 12 clear items. A second study confirmed the validity of this factorial structure and showed that *k* and mastery-approach goals were the only significant predictors of autonomous motivation regarding sport sciences studies, and that *k* was the only predictor of academic achievement in these studies. These findings support the validity of the AASQ, an instrument that now enables new types of research on approach and avoidance dynamics.

## Introduction

A dynamical model of approach and avoidance in achievement contexts has been recently proposed by Gernigon et al. (2015). This model, that borrows a dynamical systems approach to social psychology (e.g., Vallacher and Nowak, 1997, 2007; Nowak and Vallacher, 1998; Guastello et al., 2009), integrates different bodies of literature pertaining to the motivational role of perceived competence (Elliot and Dweck, 2005), former and recent conceptualizations of achievement motivation (McClelland et al., 1953; Elliot, 2005) and expectancy-value models (e.g., Atkinson, 1957; Wigfield and Eccles, 2000), and the self relevance of achievement goals (e.g., Crocker and Wolfe, 2001).

According to Gernigon et al., approach and avoidance patterns emerge from the interaction of units of information pertaining to a limited number of key social-cognitive variables: competence expectancies regarding a goal to achieve, the benefit for the self that is expected in case that goal is reached, and the threat for the self that a failure regarding the goal represents. Those variables have been selected because of their high relevance to achievement motivation. On the one hand, competence expectancies—all conceptual variations included—have long been shown to be a key determinant of individuals’ motivation to achieve (e.g., White, 1959; Harter, 1978; Bandura, 1997; Elliot and Dweck, 2005). On the other hand, goals on which individuals stake their self-worth have been shown to entail the greatest—positive or negative—motivational consequences (e.g., Crocker and Wolfe, 2001; Wolfe and Crocker, 2002). Focusing on the self relevance of goals enables to parsimoniously account for predictions and findings related to disparate—from earlier to recent—conceptualizations of motivation in achievement contexts. For instance, pioneers in achievement motivation such as McClelland et al. (1953) and Atkinson (1957) had already highlighted the importance of self concerns through the anticipations of pride and shame that are associated with the motive to achieve success and the fear of failure, respectively (see Elliot and Mapes, 2005). More recently, achievement goal theorists such as Grant and Dweck (2003) found that, unlike other types of goals, ability-linked goals (i.e., aiming at validating an aspect of self) predicted a boost to performance of students who were previously experiencing success, but withdrawal and poorer performance for students who were experiencing failure.

In the dynamical model of approach and avoidance, how competence expectancies, benefit for the self, and threat for the self are supposed to interact is inspired by expectancy-value models of achievement motivation (e.g., Atkinson, 1957; Wigfield and Eccles, 2000). According to Atkinson (1957), the tendency to achievement results from the conflicting strengths of approach and avoidance trends. The approach trend consists of a combination of a person’s dispositional motive to approach success (need for achievement), the subjective probability of success, and the incentive value of success (positive). The avoidance trend consists of a combination of the person’s dispositional motive to avoid failure (fear of failure), the subjective probability of failure, and the incentive value of failure (negative). However, for the reasons mentioned above, subjective probabilities of success or failure have been translated by Gernigon et al. in terms of expected competence or incompetence, respectively, whereas incentive values of success and failure have been specified in relation to the self, that is in terms of potential benefit and threat for the self, respectively.

The dynamical model of approach and avoidance also departs from Atkinson’s (1957) model. Whereas Atkinson viewed incentive values of success and failure as bipolar constructs, consistent with Covington and Roberts’ (1994) critique of Atkinson’s model, Gernigon et al. (2015) considered the benefit and the threat for the self as independent. Moreover, in Atkinson’s conceptualization, motives to approach success and to avoid failure are individual dispositions that play a central role in the adoption of approach and avoidance patterns. However, according to the dynamical model of approach and avoidance, states of goal involvement are patterns emerging from the complexity of systems hierarchically organized in goals and sub-goals, which are themselves determined by many dispositional, contextual, and situational factors. In other words, each of the key variables of the model—namely competence expectancies, benefit for the self, and threat for the self—can be derived from dispositional, contextual, and situational levels of influence, without *a priori* prevalence of one level over the others.

The interactions among competence expectancies (*c*), benefit for the self (*b*_*s*_), and threat for the self (*t*_*s*_)—the values of which are included between 0 and 1—have been formalized according to Eq. 1 in order to determine the value of a control parameter *k* that specifies the gradient of approach (when *k* tends toward + 1) and avoidance (when *k* tends toward –1) trends.

(1) k=(c×b)s-[t×s(1-c)]

Similarly to the Atkinson’s (1957) model, the overall approach or avoidance trend results from the conflict between the approach and avoidance members of the equation. The approach member is made up of the product of a person’s competence expectancies regarding a desired end state by the benefit for the self that is expected from success. The avoidance member is made up of the product of the person’s incompetence expectancies (expressed in terms of competence, 1 *− c*) regarding the desired end state by the threat for the self that the perspective of failure represents.

As a result, Gernigon et al.’s (2015) dynamical model of approach and avoidance in achievement contexts was shown to (a) enable predictions that are consistent with the literature on achievement motivation, social comparison, and coping strategies; (b) offer parsimonious explanations for both strong evidences and inconsistencies associated with contemporary achievement goal frameworks; and (c) generate new predictions about stability and instability of motivational states of approach and avoidance. However, there is now a need to compare the predictive value of this model with that of the recent achievement goal frameworks. To date, there is no valid instrument that specifically measures the three social-cognitive variables of the model, namely *c*, *b*_*s*_, and *t*_*s*_, on which the calculation of the parameter *k* is based. To help satisfy this need, the present twofold research aimed to develop and validate a short French questionnaire measuring the variables *c*, *b*_*s*_, and *t*_*s*_—the Approach-Avoidance System Questionnaire (AASQ)—and to test its structural and theoretical validities^1^. In a first study, a preliminary version of the AASQ was created, the factorial structure of which was then tested. In a second study, the factorial structure underwent a confirmatory test and the construct validity of the latest version of the AASQ was tested by examining the convergence of its predictions with those of contemporary frameworks of achievement goals.

## Study 1

This first study aimed to create a preliminary version of the AASQ and to examine its factorial structure. A 3-factor structure, the factors of which would correspond to competence expectancies, potential benefit, and threat for the self was expected. A small number of items per factor was wanted because tracking the dynamics of motivational states requires quick administrations of the questionnaire.

### Construction of a Preliminary Version

Based on the existing literature, we first created a preliminary version of the AASQ, the items of which were selected to assess competence expectancies as well as the potential threat and benefit that a goal may represent for the self. A committee of three experts of achievement motivation (a bilingual researcher, a Ph.D. student, and a Master’s degree student) created a first set of 19 items. Six of these items addressed competence expectancies (e.g., “I think I am good enough to achieve this goal”). Three of them were adapted from Gillet et al.’s (2008) French scale of satisfaction of the fundamental needs in sport context, whereas the other three were created by the committee. Six items addressed the benefit for the self (e.g., “If I succeeded in achieving this goal, it would reinforce my own opinion of myself”). Three of them were adapted from Vallières and Vallerand’s (1990) French Self-esteem Scale, one was adapted from Ninot et al.’s (2000) French Physical Self-Perception Profile, and the remaining two were created. Seven items addressed the threat for the self (e.g., “If I failed in achieving this goal, I would have a poor self-image”). Four of them were back-translated (Brislin et al., 1973) and adapted from Conroy’s (2001) Performance Failure Appraisal Inventory, whereas the other three were created.

In order to address an achievement context, the 19 items were distributed by packs of 6 among 60 various-sport athletes engaged in regional-level competition (49 males; 11 females; *M*_age_ = 22.7; *SD* = 2.11). These athletes voluntarily and anonymously participated in this survey. They were asked to think about a specific challenging goal they were pursuing as an athlete, then to assess the clarity of the items relating to that goal on a 5-point Likert-type scale ranging from 1 (not clear at all) to 5 (very clear). All the items had an average score greater than 3.6/5, which reflects good levels of clarity. Therefore, all of them were retained for the preliminary version of the AASQ. The next step consisted of examining the factorial structure of this version and to select the most relevant items per factor.

### Examination of the Factorial Structure

#### Method

A sample of 666 undergraduates (479 males; 187 females; *M*_age_ = 19.6; *SD* = 1.54) in their first or the second academic year of sport sciences studies participated in this research devoted to the examination of the factorial structure of the AASQ. This specific population was selected because undergraduates have the common goal to become graduates and because dropping out and failure rates are higher in the first two academic years than in the third one, which favors the largest spectrum of motivational patterns from avoidance to approach.

Participants were enrolled in the study during group lessons at the faculty of sport sciences of the authors’ university. During the lesson, students were informed that their participation in the study was not compulsory and that—if they accept to participate—their data will be kept confidential. They were told that the study would in no way influence their course grade and that there are absolutely no right or wrong answer, nor is there any value judgment about their answers. After having signed an informal consent, the volunteers read the instructions that were projected on the wall screen of the classroom as well as the following sentences inviting them to focus on the goal of obtaining their first degree: “*You are currently attending a Bachelor’s program in sport and exercise sciences. This probably means that getting the first degree is a goal for you. In order to study what this goal may represent, we would like you to answer a number of items which relate precisely to that goal.”* Then they were asked to write the goal at the top of the questionnaire sheet and to answer the 19-item version of the AASQ with reference to that goal using a Likert-type scale ranging from 0 (strongly disagree) to 10 (strongly agree).

In order to test the suitability of the data for structure detection both the Kaiser-Meyer-Olkin (KMO) measure of sampling adequacy and the Bartlett’s test of sphericity were carried out. The KMO measure indicates the proportion of variance (minimum > 0.50; best if close to 1) in the variables that is accounted for by the underlying factors. The Bartlett’s test of sphericity tests the null hypothesis that the variables are unrelated (expected *p* < 0.05). Then, participants’ scores for the 19 items were submitted to an Exploratory Factor Analysis (EFA) using the principal axis factoring method of extraction with direct oblimin rotation, so as to obtain the most simple, accurate, and interpretable representation of the data by allowing the factors to correlate (e.g., Fabrigar et al., 1999; Costello and Osborne, 2005). Moreover, a parallel analysis (Horn, 1965) was conducted to determine the number of factors to retain. Parallel analysis leads to retain as many factors as there are eigenvalues calculated from the original correlation matrix to be greater than those calculated from randomly generated correlation matrices. Items were selected on the basis of their Marker Indices (MI; Gallucci and Perugini, 2007), which account for their usefulness in representing factors by weighting both their primary and secondary loadings. As recommended by Gallucci and Perugini, a cut-off of 0.40 was adopted to estimate an MI as good. Cronbach’s (1951) coefficients alpha were calculated to account for the internal consistencies of the subscales corresponding to the factors. The EFA and the parallel analysis were processed using the SPSS Statistics 21© program enriched with the parallel analysis module developed by O’Connor’s (2000) for SPSS.

### Results

The KMO measure of sampling adequacy was excellent (0.88) and the Bartlett’s test of sphericity was significant (*p* < 0.0001). Therefore, the suitability for factor analysis of the 19-item data was supported. The parallel analysis revealed six eigenvalues from the factor analysis to be greater than their corresponding random eigenvalues. However, three of them were greater than 1 (ranging from 1.55 to 4.57), while the other three were markedly below 1 (ranging from 0.16 to 0.49). Parallel analysis of principal factor analysis is known to over-extract factors (Buja and Eyuboglu, 1992; Glorfeld, 1995) by yielding eigenvalues for negligible factors higher than their corresponding random data eigenvalues. Therefore, as recommended by O’Connor (2000), additional procedures were used to help trim trivial factors. Both Kaiser criterion (eigenvalues ≥ 1) and Cattell’s scree test (eigenvalues above the break of their plot) led to retain three factors. These factors corresponded to the expected three subscales: *t*_*s*_, *c*, and *b*_*s*_ (in descending order of eigenvalues). Except one item, which was first removed, all the items displayed MIs above 0.40 (ranging from 0.51 to 0.87) for the factors on which they were expected to load exclusively and negative MIs for the other two factors. Then, in order to create a short questionnaire, only the best four items (i.e., with the highest MIs) were retained for each factor. The scores for the remaining 12 items were submitted to a final EFA iteration which confirmed that these items did exclusively load on their respective factors. Table 1 presents the 12 selected items as well as the results of the EFA. The internal consistencies of the three subscales were good with all coefficients alpha above 0.70. Low and moderate positive correlations were found between the scales *c* and *b*_*s*_ and between the scales *b*_*s*_ and *t*_*s*_, respectively. Descriptive statistics, internal consistencies, and intercorrelations for the AASQ scales are presented in Table 2.

**TABLE 1:** Items (French and English free translation), factor loadings and eigenvalues per factor resulting from the exploratory factor analysis with direct oblimin rotation (*n* = 666).

**Items**	**Factors**		
	**I (*t*_*s*_)**	**II (*c*)**	**III (*b*_*s*_)**
Si j’échouais dans l’atteinte de ce but, l’opinion que j’ai de moi-même en prendrait un coup. *If I failed in reaching that goal, my opinion about myself would take a hit.*	0.86	0.07	0.32
Si j’échouais dans l’atteinte de ce but, j’aurais une mauvaise image de moi-même. *If I failed in reaching that goal, I would get a poor image of myself.*	0.84	0.01	0.34
Si j’échouais dans l’atteinte de ce but, je perdrais de l’estime de moi-même. *If I failed in reaching that goal, my self-esteem would be impaired.*	0.83	0.06	0.30
Si j’échouais dans l’atteinte de ce but, cela me ferait douter de ma valeur. *If I failed in reaching that goal, it would make me doubt my worth.*	0.69	–0.01	0.34
Je me sens à la hauteur de la tâche. *I feel up to the task.*	0.04	0.89	0.15
Je pense que je suis suffisamment bon(ne) pour atteindre ce but. *I think I am good enough to achieve that goal.*	0.03	0.84	0.12
Je me sens capable d’atteindre ce but. *I feel able to achieve that goal.*	0.03	0.81	0.14
J’estime être en mesure de répondre aux exigences requises pour atteindre ce but. *I believe I can meet the requirements to achieve that goal.*	0.04	0.80	0.17
Si je réussissais à atteindre ce but, ça me donnerait une bonne image de moi-même. *If I succeeded in achieving that goal, it would give me a good image of myself.*	0.33	0.19	0.74
Si je réussissais à atteindre ce but, ça renforcerait l’opinion que j’ai de moi-même. *If I succeeded in achieving that goal, it would enhance the opinion I have of myself.*	0.33	0.04	0.71
Si je réussissais à atteindre ce but, je m’en sentirais grandi(e). *If I succeeded in achieving that goal, I would feel myself more worthy.*	0.26	0.10	0.57
Si je réussissais à atteindre ce but, je serais fier(e) de moi. *If I succeeded in achieving that goal, I would be proud of myself.*	0.15	0.11	0.54
Eigenvalues	2.92	2.85	2.17

**c*, competence expectancies; *b*_*s*_, benefit for the self; *t*_*s*_, threat for the self. In EFA with oblique rotation, the eigenvalues cannot be added to calculate the total variance that is explained by the factors because of the non-orthogonality of these factors.*

**TABLE 2:** Descriptive statistics, internal consistencies, and intercorrelations for the AASQ scales.

	***M***	***SD***	**Cronbach’s** α	***c***	***b*_*s*_**	***t*_*s*_**
*c*	7.61	1.52	0.90	–		
*b*_*s*_	7.53	1.58	0.73	0.14^∗∗∗^	–	
*t*_*s*_	5.37	2.50	0.88	0.04	0.35^∗∗∗^	–

**c*, competence expectancies; *b*_*s*_, benefit for the self; *t*_*s*_, threat for the self. ^∗∗∗^*p* < 0.001.*

### Discussion

The aim of this first study was to create a preliminary version of the AASQ and to test its factorial structure. The AASQ was found to display a satisfactory 3-factor structure with consistent subscales made of clear items of competence expectancies, potential benefit for the self, and threat for the self. Although the positive correlation that was found between the scales *c* and *b*_*s*_ is significant, caution should be exercised before interpreting this finding as meaningful. Indeed, this correlation was very low and was obtained with a large sample of participants, whereas large samples are known to artificially and dramatically reduce *p* values (e.g., Nuzzo, 2014). The same reasoning may apply to the moderate positive correlation that was found between the scales *b*_*s*_ and *t*_*s*_. Nevertheless, it is interesting to note that *b*_*s*_ and *t*_*s*_ did not appear as bipolar constructs. This finding is consistent with Gernigon et al.’s (2015) view that perceptions of benefit and threat for the self may be simultaneously high or low, contrary to Atkinson’s (1957) view that incentive values of success and failure are bipolar (see also Covington and Roberts’, 1994, for a critique of Atkinson’s point of view).

Finally, the reduced number of items of the AASQ—four per subscale—is suited for quick administrations of the questionnaire. However, the theoretical validity of the AASQ remains to be tested, especially through the examination of the convergences between the predictive value of the AASQ and that of contemporary models of achievement goals.

## Study 2

This second study aimed to confirm the factorial structure of the 12-item version of the AASQ and test its construct validity by examining its convergence with a contemporary achievement goals framework, namely Elliot et al.’s four-goal framework (Elliot and McGregor, 2001; Elliot and Murayama, 2008), in predicting autonomous motivation.

Over the last three decades, the achievement goal approach to achievement motivation evolved from a starting 2-goal conceptualization (e.g., Nicholls, 1984; Dweck, 1986) to 3-goal (Elliot and Church, 1997), 4-goal (Elliot and McGregor, 2001; Elliot and Murayama, 2008), and even 6-goal (Elliot et al., 2011) frameworks. To date, given the paucity of research having addressed the 6-goal model, the model that has accumulated most empirical validations remains the 4-goal model. According to this model, the four goals that are considered to entail important motivational consequences are: Mastery-Approach (MAp) goals that consist in trying to master a task or progress in it; Mastery-Avoidance (MAv) goals that consist in aiming at not doing mistakes or doing worse than previously; Performance-Approach (PAp) goals that consist in trying to outperform others; and Performance-Avoidance (PAv) goals that consist in aiming at not being outperformed by others. The literature (see Hulleman et al., 2010; Van Yperen et al., 2014, 2015, for meta-analytic reviews) has generally shown that MAp and PAp goals are motivationnaly adaptive, whereas PAv goals are maladaptive. MAv goals have however been found to entail mixed consequences, because—according to Elliot and McGregor (2001)—they combine adaptive criteria (i.e., self-referenced) of success with a maladaptive focus on a possible negative valence of the outcome (e.g., failure).

Self-determined motivation includes several of the constructs of motivation that range along a continuum extending from the absence of motivation to the most autonomous form of motivation (e.g., Ryan and Connell, 1989; Howard et al., 2017; Ryan and Deci, 2017). From the least autonomous to the most autonomous forms of motivation of the continuum, Ryan and Connell (1989) identified amotivation (i.e., absence of motivation due to a lack of perceived contingencies between outcome and behavior), external regulation (i.e., motivation extrinsically controlled by perspectives of rewards or punishments), introjected regulation (i.e., external motivation partially internalized by perspectives of self-evaluation), identified regulation (i.e., extrinsic motivation also autonomously driven by perceived meaningfulness of activity), integrated regulation (i.e., motivation mainly autonomously driven by perceived congruence of activity with one’s identity), and intrinsic motivation (i.e., type of motivation activated for the sake and the enjoyability of activity). Three forms of intrinsic motivation were then distinguished by Vallerand et al. (1992): Intrinsic motivation to know (i.e., internal type of regulation activated by pleasure resulting from learning), intrinsic motivation to accomplish (i.e., internal type of regulation activated by pleasure resulting from accomplishing or creating something), and intrinsic motivation to experience stimulation (i.e., internal type of regulation activated by pleasure resulting from agreeable sensations). Along this continuum, controlled motivation is usually considered to include types of regulation ranged from amotivation to introjected regulation, whereas autonomous motivation is considered to include those ranged from identified regulation to intrinsic motivation (e.g., Vallerand and Bissonnette, 1992; Fortier et al., 1995; Guay and Vallerand, 1997; Vallerand et al., 1997).

The most autonomous type of motivation, intrinsic motivation, has been found to be positively related to MAp goals (e.g., Butler, 1987; Duda et al., 1995; Elliot and Harackiewicz, 1996; Brunel, 1999; Elliot and Murayama, 2008; Hulleman et al., 2008), but not to MAv goals (e.g., Elliot and Murayama, 2008). Inconsistent findings have been reported regarding whether the other types of goals are related to intrinsic motivation (e.g., Elliot and Harackiewicz, 1996; Elliot and Church, 1997; Skaalvik, 1997;, Study 2) or not (e.g., Elliot and Church, 1997; Elliot and Murayama, 2008; Hulleman et al., 2008; Elliot et al., 2011; Mascret et al., 2015). More generally, autonomous forms of motivation were found to be positively predicted by MAp goals (Ntoumanis, 2001; Standage et al., 2003b) and by motivational climates promoting such goals (Standage et al., 2003a, b). As regards academic performance, the recent meta-analytic review conducted by Van Yperen et al. (2014) showed that, generally, performance was positively related to both types of approach goals. Based on this body of literature, we hypothesized that *k*—which results from Eq. 2 and determines the tilt of the approach-avoidance attractor landscape—should positively predict autonomous motivation, as MAp goals should do. The parameter *k* should also positively predict exams performance, as both MAp and PAp should do.

### Method

#### Participants

A new sample of 400 students in sport sciences (307 males; 93 females; *M*_age_ = 19.8; *SD* = 1.6) in their first academic semester participated in this study. The reasons why this specific population was targeted were the same as for Study 1.

#### Procedure

The procedure resembled the last part of Study 1. However, it differed in that, in addition to the items of the AASQ, items addressing achievement goals and self-determined motivation were to be answered.

#### Measures

The three variables, *c*, *b*_*s*_, and *t*_*s*_, enabling the calculation of the control parameter *k* were measured with the 12-item version of the AASQ that was obtained in Study 1. The scores of *c*, *b*_*s*_, and *t*_*s*_, were calculated by averaging the scores of their corresponding items, then by dividing the averaged scores by 10 so that they are included between 0 and 1 before entering the calculation of *k* through Eq. 1.

Achievement goals were measured using Riou et al.’s (2012) French Achievement Goals Questionnaire for Sport and Exercise (FAGQSE) that was adapted to the field of education by changing the reference to sport or exercise in the instructions to an explicit reference to sport sciences studies. This instrument measures MAp goals (e.g., “My goal is to improve as much as possible.”), MAv goals (e.g., “I am striving to avoid doing things badly.”), PAp goals (e.g., “My goal is to perform better than others.”), and PAv goals (e.g., “My aim is to avoid performing worse than others.”) following Elliot and Murayama’s (2008) psychometric updates that consist of assessing goals directly and exclusively, that is without any reference to goal motives and to terms that can be indifferently applicable to several types of goals, without pitting one goal against another, without any affective content, and without focusing on extreme populations of potential winners or losers (performance goals). Items were rated on a 5-point Likert-type scale ranging from 1 (completely disagree) to 5 (completely agree). The score of each type of goal was calculated by averaging the scores of its corresponding items.

Motivation for sport sciences studies was measured using Vallerand et al.’s (1989) French Motivation Scale in Education. This questionnaire includes the scales of the different forms of motivation ranging along the self-determination continuum, except integrated regulation which was found to be hardly distinguishable from identified regulation (Vallerand et al., 1989; Howard et al., 2017). Items were propositions of answers to the question: “Why are you studying in sports sciences?”. Examples of items are: “Honestly I don’t know; I really feel that I’m wasting my time in sport sciences.” (amotivation); “To get a more prestigious job later on.” (external regulation); “To prove to myself that I can do better than just a high-school degree.” (introjected regulation); “Because it might allow me to enter the job market in a field I like.” (identified regulation); “Because I feel pleasure and satisfaction in learning new things.” (intrinsic motivation to know); “For the pleasure I feel in surpassing myself in my studies.” (intrinsic motivation to accomplish); “Because I get my kicks out of reading on various interesting subjects.” (intrinsic motivation to experience stimulation). Items were rated on a 7-point Likert-type scale ranging from 1 (does not correspond at all) to 7 (corresponds exactly). Mean scores for each subscale were used to calculate an autonomy index that is based on the substraction of the weighted sum of the controlled forms of motivation from the weighted sum of the autonomous forms of motivation (for instances, see Grolnick and Ryan, 1987; Vallerand and Bissonnette, 1992; Fortier et al., 1995; Vallerand et al., 1997; Senécal et al., 2003; Boiché and Sarrazin, 2009; for various declensions of this index). Consistent with the latest refinements of self-determination theory, the formula of the autonomy index was [2 × (intrinsic motivation to know + intrinsic motivation to accomplish + intrinsic motivation to experience stimulation)/3 + identified regulation] − [(introjected regulation + external regulation)/2 + (2 × amotivation)].

Exams performance was measured through the average grade obtained by the students at the exams of the ongoing semester.

#### Analyses

Participants’ scores for the 12 items of the AASQ were submitted to a Confirmatory Factor Analysis (CFA) that was processed using SPSS AMOS 21© program. A covariance matrix was used to seek for a solution based on maximum-likelihood estimation. In the loading matrix, error covariances were constrained to zero, but covariances between the latent factors *c*, *b*_*s*_, and *t*_*s*_ were allowed. The fit indices that were considered were the χ^2^/*df* ratio, the Comparative Fit Index (CFI), the Tucker-Lewis Index (TLI), the Root Mean Square Error of Approximation (RMSEA), and the RMSEA’s 90% confidence interval. Consistent with Kline’s (2005) recommendations, the criteria used to support a good model fit were: χ^2^/*df* < 3.00; CFI > 0.90; TLI > 0.90; and RMSEA < 0.08. As recommended by Chen et al. (2008), the lower and upper bounds of the RMSEA’s 90% confidence interval should be lower than 0.05 and 0.1, respectively. Cronbach’s coefficients alpha were also calculated to test the internal consistencies of the subscales again.

Finally, after having conducted preliminary correlation analyses between *k* and achievement goals, two stepwise regression analyses were processed to detect—among *k* and achievement goals—the predictors of autonomous motivation for sport sciences studies and of exams performance.

### Results

Regarding the confirmation of the factorial structure, the CFA revealed good fit indices: χ^2^/*df* ratio = 2.11; CFI = 0.98, TLI = 0.97; RMSEA = 0.05; lower and upper bounds of the RMSEA’s 90% confidence interval = 0.04 and 0.07, respectively. Coefficients alpha were also good with values of 0.91 for *c* scale, 0.75 for *b*_*s*_ scale, and 0.87 for *t*_*s*_ scale.

The preliminary correlation analyses conducted between *k* and achievement goals showed the *k* was positively related to MAp goals (*r* = 0.31, *p* < 0.001), MAv goals (*r* = 0.31, *p* < 0.001), PAp goals (*r* = 0.17, *p* < 0.001), and PAv goals (*r* = 0.11, *p* < 0.05).

As regards the examination of the convergence of the AASQ with other motivational constructs, regressing the autonomy index on *k*, MAp goals, MAv goals, PAp goals, and PAv goals revealed that this index was significantly predicted only by MAp goals (β = 0.41; *p* < 0.001; specific *R*^2^ = 0.22) and *k* (β = 0.20; *p* < 0.001; specific *R*^2^ = 0.04). The two-variable model that was obtained thus accounted for 26% of the variance of the autonomy index.

Regressing exams performance on *k* and the four achievement goals revealed that *k* was the only predictor of exams performance (β = 0.14; *p* < 0.001; *R*^2^ = 0.02).

### Discussion

The first aim of this second study was to confirm the 3-factor structure of the 12-item version of the AASQ that was found in Study 1. The second aim was to test the construct validity of this version by examining the convergences between its predictive value and that of Elliot et al.’s four-goal framework (Elliot and McGregor, 2001; Elliot and Murayama, 2008) regarding autonomous motivation and exams performance. Autonomous motivation was expected to be positively predicted by both MAp goals and the parameter *k*, whereas exams performance was expected to be positively predicted by MAp goals, PAp goals, and *k*.

Results from the CFA supported the adequacy of the model with the data collected from a new sample of students. The good internal consistency of each of the three subscales of the AASQ was also supported.

The preliminary correlation analyses revealed moderate positive relationships between *k* and mastery (i.e., both MAp and MAv) goals and low positive relationships between *k* and performance (i.e., both PAp and PAv) goals. We did not express any hypotheses regarding these relationships because *k* is assumed to account for either adaptive approach patterns or maladaptive avoidance patterns, whereas in Elliot and McGregor’s (2001) framework, approach and avoidance belong to the very definition of goals regardless of their resulting motivational patterns, which may sometimes be similar (see Elliot and McGregor, 2001, as well as Elliot and Moller, 2003, for discussions about the issue of mixed consequences of different goals). Nevertheless, the present correlations suggest that mastery goals are most associated with adaptive approach trends.

Regarding the examination of the convergence of the AASQ with other motivational constructs, as expected, both MAp goals and *k* were found to positively predict the autonomy index. These parallel relationships support the well-known predictive value of MAp goals regarding autonomous motivation (e.g., Elliot and McGregor, 2001; Ntoumanis, 2001; Elliot and Murayama, 2008), but most importantly, they provide a first evidence of the relevance of *k* in accounting for adaptive patterns of motivation.

Consistent with our second hypothesis, *k* did positively predict exams performance. However, MAp and PAp goals—which were expected to do so—did not. In their meta-analytic review of achievement goal measures, Hulleman et al. (2010) found that the relationships between MAp goals and performance depend on how MAp items are labeled. For instance, MAp goals positively relate to performance when their items refer to goal-related affects or interest, but not when their items explicitly refer to goals. In Riou et al.’s (2012) questionnaire used in the present research to measure achievement goals, all the items are labeled following Elliot and Murayama’s (2008) recommendation to exclusively refer to goals. This might explain why we found no relationship between MAp goals and exams performance. However, this finding as well as Hulleman et al.’s is not consistent with the well-documented goal setting literature according to which goals defined in specific terms positively influence performance (e.g., Locke and Latham, 1990). Future research is necessary to disentangle this inconsistency. The fact that the relationship between achievement goals and performance depends on the wording of goal items also applies to PAp goals. PAp goals were generally found to be positively related (Hulleman et al., 2010) or more strongly positively related (Van Yperen et al., 2014) to performance when their items are normatively referenced, that is, in terms of social comparison as in the present research. However, in an important series of studies aimed at testing the effects of different operationalizations of achievement goals, Grant and Dweck (2003) found that normative goals have no effect on the performance of students facing challenges. It remains to be seen whether the perception of challenge that was elicited in Grant and Dweck’s research is the key variable that might explain this lack of effect. With respect to our present study, it is likely that obtaining a Bachelor’s degree is perceived quite difficult for students who are only in their first academic semester. However, in the absence of a direct measure of perceived difficulty or challenge, this explanation remains speculative.

The finding that *k* predicts exams performance completes the finding that *k* predicts autonomous motivation. As a result, by combining few key social-cognitive variables such as *c*, *b*_*s*_, and *t*_*s*_, the parameter *k* intended to reflect approach and avoidance motivations does account for motivation in achievement contexts.

## General Discussion

According to Gernigon et al.’s (2015) dynamical model of approach and avoidance motivations in achievement contexts, the combination of a limited number of social-cognitive variables—namely competence expectancies, benefit for the self, and threat for the self—is sufficient to generate a control parameter *k* of approach and avoidance motivations. The present research aimed to develop and validate a self-report questionnaire—the AASQ—that measures these social-cognitive variables and therefore enables the calculation of *k*.

The AASQ is a 3-factor questionnaire, the three subscales of which are consistent and reflect competence expectancies, potential benefit for the self, and threat for the self. The parameter *k* was found to successfully mimic the well-known predictive property of MAp goals regarding autonomous motivation and to predict academic achievement. As a result, the AASQ is a reliable and valid instrument, the shortness of which enables easy and quick administrations, so as to track the dynamics of approach and avoidance motivations in various achievement contexts such as academics, the work place, and sport. However, it should be recognized that the current validation relies on two samples of participants that are extremely unbalanced with respect to gender. The proportions of men (74%) and women (26%) who participated in this research are perfectly representative of those observed throughout France in sports sciences studies^1^. This limitation of the generalizability of the present results across genders should be overcome in future research by addressing gender-balanced populations.

Beyond the psychometrical qualities of the AASQ that have been here shown, further testament of the theoretical relevance of this instrument still needs to be provided. For instance, empirical research should be conducted to evidence the sensitivity of the AASQ to experimental conditions that are assumed to elicit states of approach or avoidance goal involvement. The predictive value of the AASQ regarding achievement motivation should then be compared to that of classical four- (Elliot and McGregor, 2001; Elliot and Murayama, 2008) or six- (Elliot et al., 2011; Mascret et al., 2015) goal questionnaires. For the long term, an interesting challenge would be to test—using the AASQ—whether the dynamical model of approach and avoidance motivations in achievement contexts can resolve the inconsistencies found in research on achievement goals.

The AASQ is also useful for applied purposes. Teachers, managers, coaches, parents, and more generally, every person in charge of encouraging people’s achievement motivation can use the AASQ to track the ebb and flow of motivational states of approach and avoidance. Furthermore, they can use the information provided by the AASQ about the ups and downs of the key social cognitive determinants of such states—namely *c*, *b*_*s*_, and *t*_*s*_—to accurately orient their intervention.

## Data Availability Statement

The datasets generated for this study are available on request to the corresponding author.

## Ethics Statement

According to the French legislation (Decree No. 2017-884 of May 9, 2017) and the authors’ Institutions’ guidelines, an ethics approval was not required for this study as experiments in human and social sciences are considered out of the scope of research involving the human person requiring the authorization from committees for the protection of persons. Such is the case for the non-invasive present research which is only based on questionnaire investigations. These investigations were however conducted according to the ethical principles of human experimentation advocated in the Helsinki Declaration. Thus, all subjects gave informed written consent and were free to leave the study at any time without having to justify their abandonment.

## Author Contributions

AT participated in the conception of the purpose of this research, computed the statistics of Study 2, and wrote the manuscript. CK and CM also participated in the conception of the research purpose, collected the data, and computed the statistics of Study 1. CG supervised this research, proposed its purpose, defined its design, and corrected the writing of the manuscript.

## Conflict of Interest

The authors declare that the research was conducted in the absence of any commercial or financial relationships that could be construed as a potential conflict of interest.

## Footnotes

1 https://c3d-staps.fr/les-staps/filles-et-garcons-en-staps
